# ADRB2 Arg16Gly Polymorphism and Pulmonary Function Response of Inhaled Corticosteroids plus Long-Acting Beta Agonists for Asthma Treatment: A Systematic Review and Meta-Analysis

**DOI:** 10.1155/2018/5712805

**Published:** 2018-01-21

**Authors:** Xi Wang, Qian Li, Ruming Liu, Jin He, Di Wu, Yun Wang, Jun Zhang

**Affiliations:** The Department of Clinical Pharmacy, The First Affiliated Hospital of Kunming Medical University, Kunming, China

## Abstract

**Background:**

The beta-2 adrenergic receptor (ADRB2) Arg16Gly polymorphism may alter the bronchodilation response to long-acting beta2-agonists, thereby influencing the clinical effectiveness of LABAs plus corticosteroids (ICS) treatment. But the results of individual studies are inconclusive.

**Methods:**

A systematic search was conducted in PubMed, Embase, the Cochrane Database, WHO International Clinical Trials Registry Platform, Chinese National Knowledge Infrastructure, Wanfang, Chinese BioMedical Literature Database, and VIP databases. The meta-analysis was performed with RevMan statistical software (version 5.2), and potential publication bias was estimated by Egger's test using STATA (version 12.0), with *p* < 0.05 indicating significant publication bias.

**Results:**

We found 5 cohort studies with a total of 632 patients and included them in the meta-analysis. There are no significant differences in pulmonary function response between patients with the Arg/Arg and Arg/Gly phenotype (SMD −0.04, 95% CI −0.45 to 0.37; *p*=0.84). There were also no significant differences in the pulmonary function response between patients with the Arg/Arg and Gly/Gly phenotype (MD −0.03, 95% CI −0.07 to 0.02; *p*=0.28).

**Conclusions:**

Our systematic review and meta-analysis suggest that ADRB2 Arg16Gly polymorphism is not associated with pulmonary response to asthma treatment with ICS plus LABAs.

## 1. Introduction

According to the World Health Organization (WHO), asthma is an important noncommunicable disease with 235 million patients worldwide in 2013. The combination of inhaled corticosteroids (ICS) and long-acting beta2-agonists (LABAs) is a widely used maintenance therapy for asthma. Recent studies have revealed that beta-2 adrenergic receptor (ADRB2) polymorphism may mediate the bronchodilation response to LABA, thereby influencing the clinical effectiveness of combination treatment [1]. However, reports of the effect of Arg16Gly polymorphism on ADRB2 function are not conclusive [2, 3]. Some found no significant differences in the improvement of pulmonary function in asthmatic patients with different ADRB2 genotypes [4, 5]. Others found that the effectiveness of ICS and LABAs treatment was increased in asthma patients with the Arg/Arg phenotype [6]. We performed a systematic review and meta-analysis to investigate the association of Arg16Gly polymorphism of ADRB2 and pulmonary function response of LABAs plus ICS combination treatment in asthma patients.

## 2. Methods

We followed the STREGA (Strengthening the REporting of Genetic Association) guidelines to review the methods and results of studies reporting on the influence of ADRB2 Arg16Gly polymorphism on the pulmonary function response of ICS and LABAs treatment of asthma patients.

### 2.1. Literature Search

Two investigators (Wang and Li) independently searched PubMed, Embase, the Cochrane Database, WHO International Clinical Trials Registry Platform, Chinese National Knowledge Infrastructure, Wanfang, Chinese BioMedical Literature Database, and VIP databases. The last search was conducted on June 31, 2016. The searches followed the following strategy: (salmeterol OR formoterol OR LABAs OR long-acting beta 2 agonists) AND (adrenergic receptor OR ADRB2 OR adrenoceptor beta 2) AND (polymorphism OR SNP^∗^ OR variant OR mutation) AND asthma.

### 2.2. Study Selection

**Figure 1:**
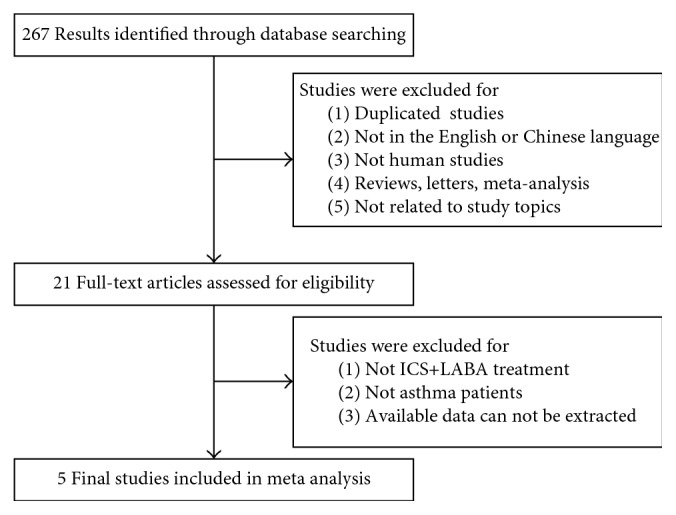
Flow chart of article selection.

A flow chart of study selection is presented in Figure 1. Studies were selected independently by two investigators (Wang and Li) and were included if they met the following criteria:

1. If they evaluated the relationship between ADRB2 Arg16Gly polymorphism and fixed dose ICS-LABAs combination treatment in asthma
2. If they included data on dosage and duration of treatment, genotype distribution, and measurement of pulmonary function
3. If they were cohort or case-control studies in humans

Studies were excluded if they met any of the following criteria:

1. If they were reviews, comments, letters, or editorials
2. If they were duplicated or overlapping studies
3. If they were not published in the English or Chinese language

### 2.3. Data Extraction and Quality Assessment

Data on first author's name, year of publication, ethnicity of participants, number of participants, sex ratio, age, asthma treatment, pulmonary function measurements, and genotyping measurements were extracted from the included study.

The primary outcomes were changes in pulmonary function from baseline, including forced expiratory volume in the first second (FEV1), peak expiratory flow (PEF), and forced vital capacity (FVC).

A quality assessment of the studies was performed independently by two observers using the Newcastle-Ottawa Scale (NOS) criteria, which include scoring of the method of group selection, comparability of participants, and clinical outcomes. The highest score is 9, and a score ≥ 6 indicates high methodological quality. Differences were resolved by discussion.

### 2.4. Statistical Analysis

The meta-analysis was performed with RevMan statistical software (version 5.2), and potential publication bias was estimated by Egger's test using STATA (version 12.0), with *p* < 0.05 indicating significant publication bias. The mean difference (MD) was calculated if the studies being compared used the same pulmonary function measurements, otherwise the standardized mean difference (SMD) was calculated, both with 95% confidence intervals (CIs). Study heterogeneity was assessed by the Cochran's *Q* and *I*^2^ tests.

## 3. Results

### 3.1. Studies Selection and Characteristics

267 articles were identified by electronic and manual searches from the databases mentioned, and 246 articles were excluded based on the titles and abstracts, then 16 articles were excluded after reviewing the full text. Finally, 5 cohort studies were included in the meta-analysis, ranging from 2006 to 2015 [4, 5, 7–9]. A total of 632 patients were included: 176 patients were with ArgArg phenotype, 265 patients were with ArgGly phenotype, and 184 patients were with GlyGly phenotype.

Two studies were carried out among Chinese Han people [7, 9], one study was carried out among Iranian [8], and other studies were carried out among mixed population or population without specification [4, 5].

In three studies, the subjects were entered two or sixteen weeks run-in period, only using albuterol or ipratropium bromide as needed to relieve asthma symptoms, then entered to the treatment of FSC period [4, 5, 7]. In two studies, the subjects were directly entered to the treatment of FSC period [8, 9].

One study examined the changes of morning PEF and FEV1 from baseline at the end of 12 weeks of treatment with FSC, 250/50 *μ*g diskus twice daily.

One study examined the changes of FEV1% predicted and FEV1/FVC from baseline at the end of 4 weeks of treatment with FSC, 250/50 *μ*g diskus twice daily. One study examined the changes of morning PEF, afternoon PEF, and predose FEV1 from baseline at the end of 16 weeks of treatment with FSC, 100/50 *μ*g diskus twice daily. One study examined the changes of morning PEF and FEV1 from baseline at the end of 12 weeks of treatment with FSC, 100/50 *μ*g diskus twice daily. One study examined the changes of PEF, FEV1, and FVC from baseline at the end of 16 weeks of treatment with FSC, 100/25 *μ*g diskus twice daily and albuterol as needed. There were no significant differences in baseline pulmonary function characteristics across the Arg16Gly genotypes in these 5 studies.

Four studies include the patients with ArgArg, ArgGly, or GlyGly phenotypes, and one study include the patients with ArgArg or ArgGly phenotypes.

Direct sequencing was conducted in one study [7], PCR-RFLP (polymerase chain reaction-restriction fragment length polymorphism) was conducted in the other two studies [8, 9], and TaqMan SNP Genotyping was conducted in one study [5]. No specific genotyping method was mentioned in one study [4]. The distribution of the Arg16Gly phenotypes in all included studies was in Hardy–Weinberg equilibrium (HWE), except one study chose ninety subjects per Arg16Gly phenotype [5].

The characteristics and methodological quality of studies are summarized in Table 1, with the first author's name, year of publication, characteristics of patients, asthma treatment, genotyping measurements, and outcome measurements. All the studies were validated by NOS quality assessment.

### 3.2. ADRB2 Arg16Gly Polymorphism and Pulmonary Function Response of Inhaled ICS plus LABAs for Asthma Treatment

**Figure 2:**
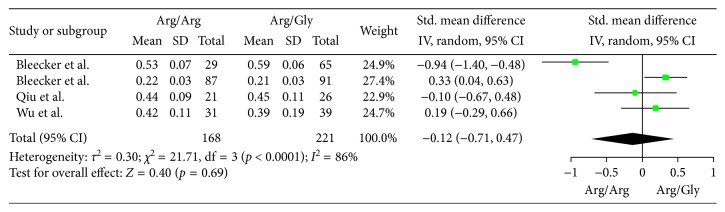
Forest plots of the association between the ADRB2 Arg16Gly polymorphism and pulmonary function response of inhaled ICS plus LABAs for asthma treatment (Arg/Arg or Arg/Gly) with 4 studies included.

**Figure 3:**
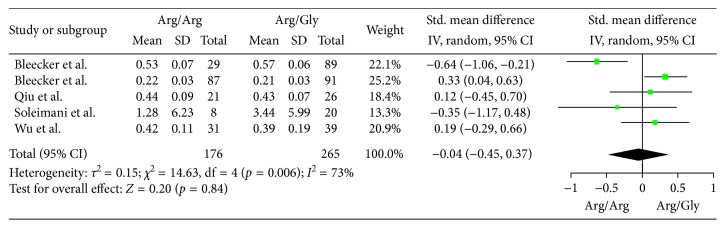
Forest plots of the association between the ADRB2 Arg16Gly polymorphism and pulmonary function response of inhaled ICS plus LABAs for asthma treatment (Arg/Arg or Arg/Gly) with 5 studies included.

The differences of pulmonary function in patients with the Arg/Arg or Arg/Gly phenotype were investigated in 5 cohort studies with a total 417 participants. The changes of FEV1(L) were the outcome measure in the 4 studies [4, 5, 7, 9], The changes of FEV1/FEC were the outcome measure in one study [8]. First, we included the 4 studies which chose FEV1 as the outcome measure to the meta-analysis with a total 389 participants, MD of FEV1 with random-effect model was performed. As shown in Figure 2, no difference of pulmonary function was found between the two phenotypes (MD −0.01, 95% CI −0.05 to 0.03; *p*=0.63). Then, we included the 5 studies to the meta-analysis with a total 417 participants, SMD with random-effect model was performed since different pulmonary functions were measured. As shown in Figure 3, no difference of pulmonary function was found between the two phenotypes either (SMD −0.04, 95% CI −0.45 to 0.37; *p*=0.84). Publication bias was assessed by Egger's test, and no significant publication bias was detected in the included studies (Table 2).

**Figure 4:**
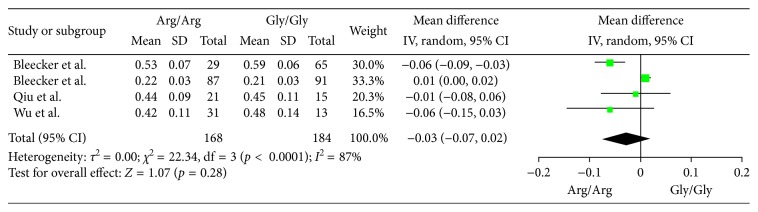
Forest plots of the association between the ADRB2 Arg16Gly polymorphism and pulmonary function response of inhaled ICS plus LABAs for asthma treatment (Arg/Arg or Gly/Gly) with 4 studies included.

The differences of pulmonary function in patients between the two phenotypes (Arg/Arg and Gly/Gly) were investigated in 4 cohort studies with a total 352 participants. FEV1(L) was the outcome measure, and MD of FEV1 with random-effect model was performed in the analysis. As shown in Figure 4, no difference of pulmonary function was found between the two phenotypes (MD −0.03, 95% CI −0.07 to 0.02; *p*=0.28). Publication bias was assessed by Egger's test, and no significant publication bias was detected in the included studies (Table 2)

## 4. Discussion

Beta2 agonist is one kind of inhaled bronchodilators, which relaxed bronchial smooth muscle by activating ADRB2 [10]. It is classified as short-acting beta agonists (SABAs) or as long-acting beta agonists (LABAs). Inhaled SABAs monotherapy is mainly used in mild or intermittent asthma patients. Since LABAs monotherapy may increase the risk of death from asthma, combination of LABAs and ICS is recommended for maintaining treatment in asthma [6]. Among around 80 identified polymorphisms in the beta-2 adrenergic receptor gene, ADRB2 Arg16Gly polymorphism is one of the most common nonsynonymous SNPs [1, 11]. ADRB2 Arg16Gly polymorphism has been shown to have functional effects in vitro. Green et al. found that human airway smooth muscle cells expressing Gly16 homozygotes resulted in enhanced ADRB2 agonist-promoted downregulation of ADRB2 compared with Arg16 homozygotes [12]. Some studies examined the clinical effect of ADRB2 Arg16Gly polymorphism on the asthmatic patient's response to LABAs plus ICS therapy, but the results were inconsistent [2, 6, 7]. Thus, this is the first systematic review and meta-analysis to estimate the effect of ADRB2 Arg16Gly polymorphism on pulmonary function in asthma patients treated with ICS plus LABAs. We did not find any significant differences of pulmonary function in patients with the Arg/Arg or Arg/Gly phenotype. Patients bearing the Arg/Arg or Gly/Gly phenotype also did not show significant different pulmonary functions. The meta-analysis results suggested that ADRB2 Arg16Gly polymorphism is not associated with pulmonary function response to asthma treatment with ICS plus LABAs.

Our analysis has limitations. Firstly, Egger's test found no significant publication bias in the included studies. However, useful data may be available in unpublished or unpresented studies and in studies that were not published in English or Chinese. Also, two potentially eligible studies were not included because of incomplete data [13, 14]. Secondly, we found significant heterogeneity among the studies, including different methods for diagnosis of asthma, different patient populations (e.g., sample size, asthma severity, age, ethnicity, and sex ratio), different fluticasone propionate and salmeterol dosage, and different treatment duration. These differences may influence the reliability of the results.

In conclusion, based on the data derived from the included studies, this systematic review and meta-analysis indicated that ArgGly polymorphism of ADRB2 may not be a predictive genetic marker of pulmonary function response to LABAs plus ICS treatment in asthma patients. Although a definitive conclusion is not possible, additional studies with larger sample sizes are warranted to address the question.

## Figures and Tables

**Table 1:** Characteristics and methodological quality of included studies.

	First author	Year	Ethnicity	Case number	Age (year)	Gender (male/female)	Main drugs and dose (*μ*g, bid)	Follow-up (week)	Outcome measure	Genotyping measurement	HWE	NOS score
1	Qiu et al. [7]	2014	Chinese Han	68	>18	no data	FSC (250/50)	12	AM PEF and FEV1	Sequencing	Yes	7
2	Soleimani et al. [8]	2013	Iranian	28	27–65	8/18	FSC (250/50)	4	FEV1% predicted and FEV1/FVC	PCR-RFLP	Yes	6
3	Bleecker et al. [5]	2009	Mixed population	270	>12	106/194	FSC (100/50)	16	AM PEF, PM PEF, and FEV1	Tag man	—	7
4	Bleecker et al. [4]	2006	Mixed population	183	>15	81/102	FSC (100/50)	12	AM PEF, FEV1	Not mentioned	Yes	7
5	Wu et al. [9]	2015	Chinese Han	83	>5	48/35	FSC (100/25)	16	FVC, FEV1, PEF	PCR-RFLP	Yes	7

**Table 2:** Egger's test.

Polymorphism	ArgArg versus ArgGly	ArgArg versus GlyGly
Numbers of studies	417	353
*p* value	0.398	0.391

*p* < 0.05, statistically significant publication bias.
