# Erratum to “ADRB2 Arg16Gly Polymorphism and Pulmonary Function Response of Inhaled Corticosteroids plus Long-Acting Beta Agonists for Asthma Treatment: A Systematic Review and Meta-Analysis”

**DOI:** 10.1155/2018/2648196

**Published:** 2018-06-05

**Authors:** Xi Wang, Qian Li, Ruming Liu, Jin He, Di Wu, Yun Wang, Jun Zhang

**Affiliations:** Department of Clinical Pharmacy, The First Affiliated Hospital of Kunming Medical University, Kunming, China

In the article titled “ADRB2 Arg16Gly Polymorphism and Pulmonary Function Response of Inhaled Corticosteroids plus Long-Acting Beta Agonists for Asthma Treatment: A Systematic Review and Meta-Analysis” [1], the Authors' Contributions section was missing and should be added as follows.
